# Programmed Cell Death Ligand 1 (PD-L1) Immunohistochemical Expression in Advanced Urothelial Bladder Carcinoma: An Updated Review with Clinical and Pathological Implications

**DOI:** 10.3390/ijms25126750

**Published:** 2024-06-19

**Authors:** Emanuela Germanà, Ludovica Pepe, Cristina Pizzimenti, Mariagiovanna Ballato, Francesco Pierconti, Giovanni Tuccari, Antonio Ieni, Giuseppe Giuffrè, Guido Fadda, Vincenzo Fiorentino, Maurizio Martini

**Affiliations:** 1 Department of Biomedical, Dental, Morphological and Functional Imaging Sciences, University of Messina, 98125 Messina, Italy; emanuelagermana@hotmail.it; 2 Department of Human Pathology in Adult and Developmental Age “Gaetano Barresi”, University of Messina, 98125 Messina, Italy; ludopepe97@gmail.com (L.P.); mariagiovannaballato96@gmail.com (M.B.); giovanni.tuccari@unime.it (G.T.); antonio.ieni@unime.it (A.I.); giuseppe.giuffre@unime.it (G.G.); guido.fadda@unime.it (G.F.); 3 Pathology Unit, Papardo Hospital, 98158 Messina, Italy; cristinapizzimenti86@gmail.com; 4 Department of Women, Children and Public Health Sciences, Catholic University of the Sacred Heart, Agostino Gemelli IRCCS University Hospital Foundation, 00168 Rome, Italy; francesco.pierconti@unicatt.it

**Keywords:** bladder carcinoma, immune system, PD-1/PD-L1 axis, immunotherapy

## Abstract

The management of advanced bladder carcinoma involves a multidisciplinary approach, but the prognosis remains poor for many patients. The immune system plays a crucial role in this disease, influencing both tumor development and response to treatment, and exploiting the immune system against the tumor can be a valuable strategy to destroy neoplastic cells. This is the biological principle underlying Bacillus Calmette–Guérin (BCG) use and, more recently, immune checkpoint inhibitors (ICIs), like PD-1 (programmed death-1)/PD-L1 (programmed death-ligand 1) inhibitors. In fact, one of the best studied immune checkpoints is represented by the PD-1/PD-L1 axis, which is a well-known immune escape system adopted by neoplastic bladder cells. PD-L1 expression has been associated with a higher pathologic stage and has shown prognostic value in bladder carcinoma. Interestingly, high-grade bladder cancers tend to express higher levels of PD-1 and PD-L1, suggesting a potential role of such an axis in mediating disease progression. Immunotherapy with PD-1 and PD-L1 inhibitors has therefore emerged as a valuable treatment option and has shown efficacy in advanced bladder cancer patients, with high PD-L1 expression levels associated with better treatment responses. Our review aims to provide a comprehensive overview of the role of PD-L1 in advanced bladder cancer, focusing on its implications for treatment decisions and the prediction of treatment response. Overall, our work aims to contribute to the understanding of PD-L1 as a predictive biomarker and highlight its role in shaping therapeutic approaches for advanced bladder cancer.

## 1. Introduction

Bladder cancer is the ninth most common malignancy worldwide and the fifth most common in developed countries [1]. According to the updated NCCN Guidelines for Bladder Cancer (version 2.2022), the clinical spectrum of bladder carcinoma can be divided into three categories that differ in prognosis, management, and therapeutic aims [1]. The first category includes non-muscle-invasive bladder cancer (NMIBC), for which therapy is directed at reducing recurrence and preventing progression to a higher grade [1]. The second category includes muscle-invasive disease (MIBC), with an increased risk of progression to a metastatic tumor, and lastly the third category consists of metastatic disease [1]. In total, 90% of all cases of bladder cancer are represented by urothelial carcinoma and its diagnosis is based on a combination of methods such as urinary cytology, urinary biomarkers, and cystoscopic examination with transurethral resection of bladder tumor (TURBT) [2,3,4,5,6,7,8]. NMIBC encompasses papillary tumors confined within the mucosa (stage Ta), tumors that invade the lamina propria (stage T1), and flat high-grade lesions known as carcinoma in situ (CIS) [9,10]. Up to 25% of patients with NMIBC progress to muscle-invasive disease after repeated recurrences and can also metastasize to the regional lymph nodes, liver, lung, bone, peritoneum, pleura, kidney, adrenal gland, intestine, mediastinum, skin, brain, heart, and testes [11,12,13].

Therapeutically, the management of advanced bladder carcinoma involves a multidisciplinary approach, including neoadjuvant chemotherapy, radiotherapy, and surgical interventions such as TURBT and cystectomy [2,14]. Approximately 20% of patients are diagnosed with muscle-invasive disease at the time of initial presentation, which will require multiple treatment modalities due to the high rates of disease recurrence, progression, and disease-specific mortality [15]. Treatment options include chemotherapy, radiation therapy, and radical cystectomy in cases of clinically localized disease and systemic chemotherapy for patients with metastatic disease. Despite this aggressive treatment approach, prognosis remains poor for many patients [2,14,15]. Moreover, up to 30–50% of patients with metastatic urothelial carcinoma are ineligible to receive cisplatin due to comorbidities [10]. Other therapeutic approaches have been adopted to exploit the host’s immune system against the tumor. The most common approach is represented by intravesical Bacillus Calmette–Guérin (BCG), that has been the gold standard for treating high-risk NMIBC for many years. The most recent therapy targeting immune checkpoint molecules, such as in urothelial carcinoma, is the PD-1 (programmed death-1)/PD-L1 (programmed death-ligand 1) axis, that have shown efficacy particularly in advanced stages. The aim of the present review is to describe the state-of-the-art knowledge regarding the prognostic and predictive role of PD-L1 expression in advanced bladder carcinoma and related treatment responses.

## 2. The Role of Immune System in Bladder Carcinoma

In solid tumors, including bladder cancer, the process of oncogenesis generally leads to genetic instability which results in the production of tumor-specific neoantigens that allow the immune response to target malignant cells [16]. Bladder carcinoma is characterized by a higher mutational burden compared to other tumors, and this heterogeneity could be due to the presence of different cancer stem cells [17]; consequently, their clones lead to a mixture of signature and discordance between global mRNA profiling and immunohistochemical features that is crucial not only for tumor cells, but also for immune responses [17]. In fact, the immune microenvironment has a central role in neoplastic processes, and the ability of the immune system to recognize and eliminate transformed cells early in the tumorigenic process is called “tumor immunosurveillance”. Immunogenicity in bladder carcinoma differs among different histological subtypes and affects both innate and adaptive immunity [17]. The immune cells involved are represented by both B and T lymphocytes (CD4, CD8, and Th1), and dendritic cells (DCs) which are resident in the bladder, and by neutrophils, macrophages, mast cells, and NK cells which are recruited from the bloodstream. An effective anti-tumor immune response will be the result of a concerted effort of antigen-presenting cells (APCs) (DCs, macrophages), lymphocytes, NK cells, and the other abovementioned immune effectors [18,19].

## 3. Mechanisms of Immune System Evasion in Bladder Carcinoma

To survive and escape from the normal immune response, tumor cells secrete various immunosuppressive and anti-apoptotic factors, such as TGF-beta, PGE2, IL-10, and IL-6, creating a highly tolerogenic microenvironment [20,21,22,23]. In addition, the tumor microenvironment is tightly linked to the accumulation of several types of immune cells with immunosuppressive phenotypes, such as myeloid-derived suppressor cells (MDSCs), tolerogenic DCs (tDCs), tumor-associated macrophages (TAMs), and regulatory T cells (T-regs) [20,21,22,23]. A highly immunosuppressive microenvironment has been described in bladder carcinoma, in which the PD-L1/PD-1 axis may play a crucial role in neoplastic immune escape [24,25,26,27]. Firstly, PD-1 was identified in 1992 as a protein involved in apoptosis, and subsequently, its role in modulating the hyper-stimulated immune system was highlighted [28]. On the other hand, PD-L1 is a 1 transmembrane protein ligand, also known as B7-H1 and it is the main ligand, functionally characterized, of the PD-1 receptor [29]. The gene PDCDL1 on chromosome 9 is responsible for the encoding, representing the third member of the B7 family proteins [29]. Its expression can be constitutive or inducible, particularly influenced by proteins such as toll-like receptors (TLRs) on APCs and activated by pathogen-associated molecular patterns [29]. PD-L1 activation depends on the ligation to PD-1 (CD279), a transmembrane receptor encoded by PDCD1 gene, physiologically expressed on lymphocytes and myeloid cells [29]. After PD-L1 binding with PD-1, the recruitment of SHP-1/SHP2 (Src homology domain containing phosphatases 1 and 2) causes a kinases cascade resulting in a general inhibition of T cells’ expansion [29]. Particularly in inflammatory conditions, PD-L1 is expressed in immune response cells, including activated T and B lymphocytes, macrophages, DCs, APCs and some epithelial cells [29,30,31,32]. Furthermore, tumor cells produce PD-L1 as an “adaptive immune mechanism” to evade anti-tumor responses; this event involves a suppressive signal to T lymphocytes, which in turn causes the immune response to diminish after interacting with either the PD-1 or B7.1 (CD80) receptors [30,31]. However, another important player in the regulation of T-cell activity is represented by CTLA-4, a crucial immune checkpoint receptor [32]. In detail, CTLA-4 has regulatory effects on T-lymphocyte activation and it is expressed on regulatory T cells and also upregulated on conventional T cells after activation [32].

Additionally, cytokines and other immunosuppressive factors secreted by tumor-recruited myeloid cells play multifaceted roles in the mechanisms of regulation of PD-L1 expression [33,34,35,36].

**Figure 1:**
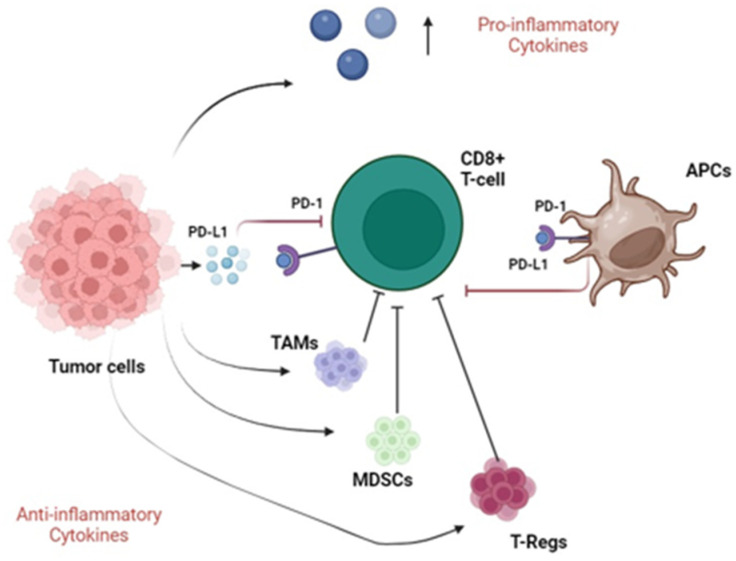
Immunosuppressive pathways in bladder cancer. Tumor cells secrete pro-inflammatory cytokines and recruit TAMs, MDSCs and T-Regs with anti-inflammatory function (black arrows), meanwhile inhibiting CD8+ T cells (blunted black arrows). Furthermore, neoplastic cells secrete PD-L1 molecules that interact with PD-1 receptors expressed by both CD8+ T cells and APCs, resulting in their inhibition (blunted red arrows). Abbreviations: TAMs, tumor-associated macrophages; MDSCs, myeloid-derived suppressor cells; T-regs, regulatory T cells; APCs, antigen-presenting cells; PD-1, programmed death-1; PD-L1, programmed death ligand 1.

Therefore, to efficiently escape the normal immune response, bladder cancer produces and secretes different cytokines with both pro- and anti-inflammatory roles. The tumor microenvironment is characterized by the presence of several immune cells with an immunosuppressive function, such as TAMs, MDSCs, and T-regs. Moreover, tumors secrete PD-L1, which interacts with the PD-1 receptor expressed by CD8+ T cells and APCs, resulting in an effective immune escape. Figure 1 illustrates the main immune escape systems adopted by bladder cancer cells.

## 4. PD-L1 Expression in Bladder Carcinoma

The assessment of PD-L1 expression in bladder carcinoma has been considered as an important part of understanding the tumor immune milieu and predicting therapeutic response [37]. In fact, PD-L1 expression in bladder carcinoma has shown a prognostic value [38] and an association with a higher pathologic stage has been demonstrated [39]. Specifically, it has been shown that high-grade bladder carcinomas express higher PD-L1 and PD-1 levels compared to low-grade ones, and PD-L1 might function as a mediator of stage progression [40,41]. However, the best application of PD-L1 evaluation in bladder carcinoma is in relation to its possible therapeutic implications. In fact, recent advances in anticancer treatments have led to the development of therapies that effectively restore the immune response against cancer cells, the so-called “immune checkpoint inhibitors” (ICIs) [41]. One of the best examples is represented by PD-1/PD-L1 inhibitors, that bind to PD-1 or PD-L1 to avoid their interaction, thus reactivating the functionality of T-lymphocytes and preventing the immunological escape of tumor cells [42]. In this regard, the evaluation of PD-L1 expression in bladder carcinoma is relevant for determining the eligibility of patients for treatment with PD-1/PD-L1 inhibitors [10]. In addition, such drugs represent an emerging treatment option for bladder carcinoma, particularly in advanced stages, and the evaluation of PD-L1 expression is performed using immunohistochemical assays. Specifically, there are several PD-L1 assays and immunohistochemical scoring systems (Table 1) performed to obtain appropriate treatment decisions for PD-1/PD-L1 targeted immunotherapy. In particular, the most commonly used scoring system in bladder carcinoma is called the combined positive score (CPS) and it is calculated by dividing the number of PD-L1 stained cells (tumor cells, lymphocytes, and macrophages) by the total number of viable tumor cells, multiplied by 100 [16]. Other scoring systems, although less utilized in bladder carcinoma, are the tumor proportion score (TPS) and immune cell (IC) score, which are individually able to measure the percentage of tumor cells showing PD-L1 expression and the area occupied by PD-L1-positive immune cells (lymphocytes, dendritic cells, macrophages, and granulocytes) as a percentage of the whole tumor area [43,44]. Of note, different cut-off values for PD-L1 status determination have been adopted (Table 1) [21,45].

The current guidelines for the treatment of advanced and metastatic bladder urothelial carcinoma suggest considering the use of ICIs as a second-line option for patients who have experienced disease progression, while on or after platinum-based therapy, regardless of their PD-L1 status, based on the results of the KEYNOTE-045 and IMvigor211 trials [46,47,48]. Specifically, in the KEYNOTE-045 phase 3 trial, 542 patients with advanced urothelial carcinoma that recurred or progressed after platinum-based chemotherapy were randomly assigned to receive pembrolizumab (a PD-1 inhibitor) or chemotherapy with paclitaxel, docetaxel, or vinflunine [47]. PD-L1 status was assessed using the PD-L1 immunohistochemistry (IHC) 22C3 pharmDx assay (Dako Agilent Technologies, Carpinteria, CA, USA), and measured using the CPS. The coprimary end points were overall survival (OS) and progression-free survival (PFS), which were assessed among all patients and among patients who had a tumor PD-L1 CPS ≥ 10. The median OS in the total population was 10.3 months (95% CI, 8.0 to 11.8) in the pembrolizumab group; meanwhile, it was 7.4 months (95% CI, 6.1 to 8.3) in the chemotherapy group (hazard ratio [HR] for death, 0.73; 95% CI, 0.59 to 0.91; *p* = 0.002). The median OS in the tumor PD-L1 CPS ≥ 10 cohort was 8.0 months (95% CI, 5.0 to 12.3) in the pembrolizumab group; meanwhile, it was 5.2 months (95% CI, 4.0 to 7.4) in the chemotherapy group (HR, 0.57; 95% CI, 0.37 to 0.88; *p* = 0.005). Moreover, there was no significant difference in PFS duration between groups in the whole population (HR for death or disease progression, 0.98; 95% CI, 0.81 to 1.19; *p* = 0.42) or among patients with a tumor PD-L1 CPS ≥ 10 (HR, 0.89; 95% CI, 0.61 to 1.28; *p* = 0.24). The pembrolizumab group had fewer treatment-related adverse events of any grade than the chemotherapy one (60.9% vs. 90.2%), as well as fewer grade 3, 4, or 5 events (15.0% vs. 49.4%) [46]. Therefore, pembrolizumab was associated with significantly longer OS and a lower risk of treatment-related adverse events than chemotherapy as a second-line treatment option for platinum-refractory advanced bladder urothelial carcinoma [47]. Similarly, in the IMvigor211 multicenter, open-label, phase 3 randomized controlled trial, 931 patients with metastatic urothelial carcinoma who had progressed after platinum-based chemotherapy were randomly assigned (1:1) to receive atezolizumab (a PD-L1 inhibitor) or chemotherapy intravenously every 3 weeks [48]. PD-L1 status was assessed using the PD-L1 Ventana SP142 immunohistochemistry assay (Ventana Medical Systems, Tucson, AZ, USA), and measured using the IC score. The scoring criteria designated tumors as IC0 (PD-L1 expression on <1% of tumor-infiltrating immune cells), IC1 (PD-L1 expression on ≥1% and <5% of tumor-infiltrating immune cells), or IC2/3 (PD-L1 expression on ≥5% of tumor-infiltrating immune cells). Randomization was stratified by PD-L1 expression, chemotherapy type (vinflunine vs. taxanes), presence or absence of liver metastases, and the number of prognostic factors [48]. OS was the primary endpoint. Although the primary analysis did not demonstrate a statistically significant longer OS for patients receiving atezolizumab versus chemotherapy, the updated OS revealed long-term durable remission [49]. With a median follow-up of 33 months, intention-to-treat (ITT) patients receiving atezolizumab had better OS rates at 24 months (23% vs. 13%) and 30 months (18% vs. 10%), as well as across PD-L1 subgroups, than those receiving chemotherapy [49]. In the ITT population, the updated OS HR (0.82, 95% CI 0.71–0.94) was similar to the OS HR obtained in the original analysis (0.85, 95% CI 0.73–0.99). The safety analysis showed that chemotherapy-treated patients experienced more grade 3/4 treatment-related adverse events (43% vs. 22%) and further adverse events resulted in treatment discontinuation (18% vs. 9%) [49]. Patients treated with atezolizumab had more adverse events of particular relevance (35% vs. 20%), which were typically grade 1–2. Overall, the trial results supported the administration of atezolizumab in platinum-treated patients with metastatic urothelial bladder carcinoma, regardless of their PD-L1 status [49].

Alternatively, in patients with metastatic/advanced bladder urothelial carcinoma who are unable to tolerate cisplatin, the Food and Drug Administration (FDA) and European Medicines Agency (EMA) have approved pembrolizumab and atezolizumab for use as first-line therapy.

In the KEYNOTE-052 trial, cisplatin-ineligible patients with locally advanced and unresectable or metastatic urothelial carcinoma received first-line pembrolizumab [50]. PD-L1 status was assessed using the PD-L1 IHC 22C3 pharmDx assay (Dako Agilent Technologies, Carpinteria, CA, USA) and measured using the CPS. Two CPS cut-offs were used: a ≥ 1 cut-off based on results from the KEYNOTE-012 trial [51] and a strongly positive CPS cut-off identified in this study. In detail, the primary endpoint was objective response rate (ORR) in patients with PD-L1-expressing tumors per response evaluation criteria in solid tumors (RECIST) version 1.1 by blinded independent central review (BICR) [50]. A total of 370 patients were enrolled and the median follow-up was 56.3 months (range 51.2–65.3 months). The confirmed ORR was 28.9% (95% confidence interval [CI] 24.3–33.8), and the median duration of response (DOR) was 33.4 months (range 1.4+ to 60.7+ months); the 36-month DOR rate was 44.8% [50]. A PD-L1 CPS ≥ 10 was associated with a higher frequency of response to pembrolizumab; 42 (38%, 95% CI 29–48) of 110 patients with a PD-L1 CPS ≥ 10 had a centrally assessed objective response. However, responses were observed in all PD-L1 expression categories, meaning that low or no PD-L1 expression did not preclude response. Most treatment-related adverse events for pembrolizumab in either study were grade 1 or 2 and manageable, which is consistent with prior reports. With approximately 5 years of follow-up, pembrolizumab monotherapy continued to demonstrate durable efficacy with no new safety signals [52].

The results of another study called IMvigor210, a single-arm, multicenter, phase 2 trial, showed excellent sustained response rates, survival, and tolerability of atezolizumab in patients with locally advanced or metastatic urothelial carcinoma who were not eligible for cisplatin [53]. Specifically, 123 patients were recruited between 9 June 2014 and 30 March 2015, out of whom 119 received atezolizumab in one or more doses. PD-L1 status was assessed using the PD-L1 Ventana SP142 immunohistochemistry assay (Ventana Medical Systems, Tucson, AZ, USA), and measured using the IC score. Scoring criteria designated tumors as IC0 (PD-L1 expression on < 1% of tumor-infiltrating immune cells), IC1 (PD-L1 expression on ≥1% and <5% of tumor-infiltrating immune cells), or IC2/3 (PD-L1 expression on ≥5% of tumor-infiltrating immune cells). The main outcome, which was evaluated in all patients and in predefined subgroups based on PD-L1 expression, was the independently verified ORR as per RECIST version 1.1 (central review). The ORR was 23% (95% CI 16 to 31) at the median follow-up of 17.2 months, the full response rate was 9% (n = 11), and all PD-L1 and poor prognostic factor subgroups showed responses [49]. The PFS rate ranged from 2.1 to 4.2 months on average and 15.9 months was the median OS period [53]. Fatigue, diarrhea, and pruritus were among the treatment-related side effects reported by 10% or more of patients; one death from sepsis related to treatment was also observed [53]. Immune-mediated events occurred in 14 patients (12%). Overall, the results of this trial supported the use of atezolizumab in untreated metastatic urothelial cancer [53].

The efficacy of atezolizumab was also demonstrated in the neoadjuvant setting of patients with MIBC, who were ineligible for or refused cisplatin-based neoadjuvant chemotherapy. In fact, the multicenter, single-arm, neoadjuvant, phase 2 study ABACUS, showed that neoadjuvant atezolizumab in MIBC is associated with clinical responses and high disease-free survival (DFS); in this trial, eighty-eight patients were given two cycles of atezolizumab before undergoing radical cystectomy [54]. PD-L1 status was assessed using the PD-L1 Ventana SP142 immunohistochemistry assay (Ventana Medical Systems, Tucson, AZ, USA), and measured using the IC score. PD-L1 positivity consisted of the staining of ≥ 5% of tumor-infiltrating immune cells. The primary clinical endpoint of the study was the pathological complete response (pCR) rate in all patients who received at least one cycle of atezolizumab and underwent radical cystectomy, or withdrew from the study for disease progression before surgery. The median follow-up period was twenty-five months (95% CI: 25–26); ninety-five individuals were administered the drug for at least one cycle; only one of them underwent a cystectomy because of an advanced illness [54]. A 31% (27/88; 95% CI 21–41) pCR rate was observed, while at two years, the OS and DFS were 77% (95% CI 68–85) and 68% (95% CI 58–76), respectively [54]. Patients who achieved a pCR had a two-year DFS of 85% (95% CI 65–94). Overall, 35 of 88 (40%) patients were positive for PD-L1 at baseline. The pCR rate in this population was 37% (95% CI 21–55%), and the 1-year relapse-free survival (RFS) rate was 75% (95% CI 53–87%). There was no significant correlation between PD-L1 expression and outcome, on either tumor-infiltrating immune cells or tumor cells (*p* > 0.05 for both). There was no significant correlation between RFS and baseline PD-L1 and tumor mutational burden (HR 0.60 [95% CI 0.24–1.5], *p* = 0.26, and 0.72 [95% CI 0.31–1.7], *p* = 0.46) [54]. Circulating tumor DNA (ctDNA) positivity values at baseline, after neoadjuvant therapy and after surgery, were 63% (25/40), 47% (14/30), and 14% (5/36), respectively [54]. RFS was related to high baseline stromal CD8+ lymphocytes (HR 0.25 [95% CI 0.09–0.68], *p* = 0.007) and high post-treatment fibroblast activation protein (HR 4.1 [95% CI 1.3–13], *p* = 0.01) [54]. Overall, the above reported data showed that neoadjuvant atezolizumab has been associated with both improved clinical responses and DFS in MIBC [54]. Moreover, bladder carcinoma patients who underwent a cystectomy after receiving immunotherapy had good long-term outcomes. It was also found that a number of biological features can aid in identifying the patients who would benefit most from this therapy [54]. 

Overall, the results of these studies show that the use of ICIs is effective regardless of PD-L1 status. Nonetheless, other studies have shown how ICI treatment can have no [55] or limited [56] efficacy in metastatic urothelial carcinoma and it has been suggested that ICIs should be recommended only for patients with a PD-L1-positive tumor [55,56,57]. In particular, KEYNOTE-361 was a phase 3 randomized open-label study for patients with untreated, locally progressed, unresectable, or metastatic urothelial carcinoma who were at least 18 years old and had an ECOG performance status of 0–2 [58]. Random assignment was used to recruit eligible patients from 201 medical centers across 21 nations. PD-L1 status was assessed using the PD-L1 IHC 22C3 pharmDx assay (Dako Agilent Technologies, Carpinteria, CA, USA), and measured using the CPS. The dual primary endpoints were PFS and OS for pembrolizumab plus chemotherapy versus chemotherapy alone for the total patient population. Stratified by choice of platinum treatment and PD-L1 CPS, 1010 patients were recruited between 19 October 2016 and 29 June 2018, receiving intravenous pembrolizumab 200 mg every three weeks for a maximum of 35 cycles, along with intravenous chemotherapy for a maximum of six cycles (n = 351), pembrolizumab alone (n = 307), or chemotherapy alone (n = 352) [58]. The median follow-up period was 31.7 months (interquartile range [IQR] 27.7–36.0) [58]. The study found that there was no significant difference in PFS between the groups receiving pembrolizumab plus chemotherapy and chemotherapy alone [58]. The median PFS was 8.3 months (95% CI 7.5–8.5) in the pembrolizumab plus chemotherapy group compared to 7.1 months (6.4–7.9) in the chemotherapy group (HR 0.78, 95% CI 0.65–0.93; *p* = 0.0033). Additionally, there was no significant difference in OS between the two groups [58]. The median OS was 17.0 months (14.5–19.5) in the pembrolizumab plus chemotherapy group and 14.3 months (12.3–16.7) in the chemotherapy group (HR 0.86, 95% CI 0.72–1.02; *p* = 0.0407). In analyses of OS with pembrolizumab versus chemotherapy (which were exploratory because the dual primary endpoints were not met), the OS was similar in both the populations as a whole (15.6 months [95% CI 12.1–17.9] with pembrolizumab vs. 14.3 months [12.3–16.7] with chemotherapy; HR 0.92, 95% CI 0.77–1.11) and in the populations with a CPS of at least 10 (16.1 months [13.6–19.9] with pembrolizumab vs. 15.2 months [11.6–23.3] with chemotherapy; HR 1.01, 95% CI 0.77–1.32). Anemia with pembrolizumab plus chemotherapy (104 [30%] of 349 patients) or chemotherapy alone (112 [33%] of 342 patients) was the most common grade 3 or 4 adverse event linked to the study treatment. Diarrhea, fatigue, and hyponatremia (each affecting four [1%] of three hundred and two patients) were the other common grade 3 or 4 adverse events. Of the 1010 participants, 6 (1%) died as a result of an adverse event linked to the study therapy (2 patients per treatment group) [58]. In conclusion, the study showed that the addition of pembrolizumab to first-line platinum-based chemotherapy did not significantly improve efficacy and should not be widely adopted for treatment of advanced urothelial carcinoma [58].

In another global, partially blinded, randomized, controlled phase 3 study, the IMvigor130 trial, 1213 patients (≥18 years) with locally advanced or metastatic urothelial carcinoma who had not received prior treatment and had an ECOG performance status of 0–2, were randomly divided into three groups (1:1:1) and assigned to receive atezolizumab plus platinum-based chemotherapy (cohort A, n = 453), atezolizumab alone (cohort B, n = 354), or placebo plus platinum-based chemotherapy (cohort C, n = 390) [57,59,60]. In addition, the patients were stratified by PD-L1 status, Bajorin score, and the investigator’s preference for platinum-based chemotherapy. PD-L1 status was assessed using the PD-L1 Ventana SP142 immunohistochemistry assay (Ventana Medical Systems, Tucson, AZ, USA), and measured using the IC score. The scoring criteria designated tumors as IC0 (PD-L1 expression on <1% of tumor-infiltrating immune cells), IC1 (PD-L1 expression on ≥1% and <5% of tumor-infiltrating immune cells), or IC2/3 (PD-L1 expression on ≥5% of tumor-infiltrating immune cells). Every three weeks, atezolizumab (1200 mg) or a placebo was injected intravenously into groups B and C. Chemotherapy consisted of 21-day cycles of intravenous carboplatin or cisplatin with gemcitabine. The study’s co-primary endpoints were investigator-assessed PFS and OS for group A against group C in the ITT population (i.e., all randomly assigned patients), as well as OS for group B versus group C, evaluated hierarchically, and then the populations with PD-L1 IC2/3 if the results from group A versus group C were significant. At the time of the final PFS analysis (31 May 2019), the median PFS in the intentional population was 8.2 months (95% CI 6.5–8.3) in group A and 6.3 months (6.2–7.0) in group C (stratified HR 0.82, 95% CI 0.70–0.96; one-sided *p* = 0.007). Overall, the observed treatment effect on PFS was consistent across subgroups. OS analysis had a one-sided *p*-value of 0.021, as indicated. After a median follow-up of 13.4 months (IQR 6.2–30.8), median OS was 16.1 months (95% CI 14.2–18.8; 336 deaths) in group A vs. 15.2 months (95% CI 13.1–17.7; 271 deaths) in group B and 13.4 months (12.0–15.3; 310 deaths) in group C (stratified HR 0.85 [95% CI 0.73–1.00]; one-sided *p* = 0.023). In patients with PD-L1 IC2/3, the median OS in the atezolizumab monotherapy group was 27.5 months (95% CI 17.7–49.4 months), compared to 16.7 months (95% CI 10.0–26.1 months) in the chemotherapy group. The stratified HR was 0.70 (95% CI 0.48–1.03). Anemia was present in 168/454 (37%) patients who received atezolizumab plus chemotherapy vs. 133/389 (34%) who received placebo plus chemotherapy, neutropenia (167 [37%] vs. 115 [30%]), decreased neutrophil count (98 [22%] vs. 95 [24%]), thrombocytopenia (95 [21%] vs. 70 [18%]), and decreased platelet count (92 [20%] vs. 92 [24%]) were the most common grade 3–4 treatment-related adverse events [60]. Serious adverse events were reported in 243 (54%) patients who received atezolizumab with chemotherapy and 196 (50%) individuals who received placebo plus chemotherapy. Nine patients (2%) died as a result of treatment in the atezolizumab plus chemotherapy group, while four patients (1%) died as a result of treatment in the placebo plus chemotherapy group. Overall, all these results demonstrated that the first-line combination of atezolizumab and platinum-based chemotherapy did not significantly increase OS in the ITT group of IMvigor130, while improving PFS rates. Interestingly, an OS improvement was observed among PD-L1 IC2/3 patients treated with atezolizumab monotherapy.

Other immunotherapy agents used as a second-line option in metastatic bladder urothelial carcinoma are represented by nivolumab (a PD-1 inhibitor) and avelumab (a PD-L1 inhibitor). Specifically, nivolumab has been recently authorized by the FDA and EMA as an adjuvant therapy after radical cystectomy for urothelial carcinoma patients who are at high risk of recurrence and for patients who exhibit high PD-L1 levels [61]. This authorization was based on the results of the CheckMate 274 trial, a phase 3 multicenter, double-blind, randomized, placebo-controlled study involving 709 patients with locally advanced bladder carcinoma [61]. MIBC patients who had undergone radical surgery were randomized to receive either nivolumab (240 mg intravenously) or placebo every two weeks for up to a year [61]. A total of 356 participants received a placebo and 353 individuals received nivolumab. PD-L1 status was assessed using the PD-L1 IHC 28-8 pharmDx platform (Dako Agilent Technologies, Carpinteria, CA, USA), and measured using the TPS. The two primary endpoints were DFS among all the patients who underwent randomization (ITT population) and among those with a TPS ≥ 1%. In the ITT population, the median DFS was 10.8 months (95% CI, 8.3 to 13.9) with placebo and 20.8 months (95% CI, 16.5 to 27.6) with nivolumab; at six months, 74.9% of patients receiving nivolumab and 60.3% receiving a placebo were alive and free of illness (HR for disease recurrence or death, 0.70; 98.22% CI, 0.55 to 0.90; *p* < 0.001). A total of 74.5% and 55.7% of patients had PD-L1 expression levels ≥ 1% (HR, 0.55; 98.72% CI, 0.35 to 0.85; *p* < 0.001) [61]. In the ITT population, the median survival free from recurrence outside the urothelial tract was 13.7 months (95% CI, 8.4 to 20.3) with placebo and 22.9 months (95% CI, 19.2 to 33.4) with nivolumab [61]. At six months, 77.0% of patients receiving nivolumab and 62.7% receiving a placebo were alive and free from recurrence outside the urothelial tract (HR for recurrence outside the urothelial tract or death, 0.72; 95% CI, 0.59 to 0.89). A total of 75.3% of patients and 56.7% of patients had a TPS ≥ 1% (HR, 0.55; 95% CI, 0.39 to 0.79). In the nivolumab group, 17.9% had treatment-related side events of grade 3 or higher, while in the placebo group, 7.2% experienced the same [61]. Globally, adjuvant nivolumab was associated with a longer DFS than placebo both in the ITT population and among patients with a PD-L1 TPS ≥ 1% [55]. Conversely, no appreciable effect was observed in patients with negative PD-L1 expression (TPS < 1%) when nivolumab was administered compared to placebo [43,61]. 

On the other side, the regulatory approval of avelumab was based on the results of the JAVELIN Solid Tumor trial [62] and the JAVELIN Bladder 100 trial [63]. The first study involved previously treated patients with advanced/metastatic urothelial carcinoma and who were given avelumab 10 mg/kg every two weeks until the disease progressed, the toxicity was intolerable, or there was drug withdrawal [62]. The endpoints were best overall response, PFS as determined by RECIST V.1.1, OS, and safety. Post hoc analyses involved ORRs in subgroups defined by known high-risk/poor prognosis features and the relationship between time to respond and outcome. PD-L1-positive status was defined using a cut-off of ≥5% expression on tumor cells, using the PD-L1 IHC 73-10 pharmDx platform (Dako Agilent Technologies, Carpinteria, CA, USA). The drug was administered to 249 patients, and 242 of them had previously received platinum-based chemotherapy. The treatment duration was 2.8 months (range 0.5–42.8) and the follow-up period was 31.9 months (range 24–43). Complete response was in 4.1% and partial response was in 12.4% of the cases; the confirmed ORR was 16.5% (95% CI, 12.1% to 21.8%). Median response lasted 20.5 months (95% CI 9.7 months to not estimable). The 12-month PFS rate was 16.8% (95% CI 11.9% to 22.4%), and the median PFS was 1.6 months (95% CI 1.4 to 2.7 months). The 24-month OS rate was 20.1% (95% CI 15.2% to 25.4%), and the median OS was 7.0 months (95% CI 5.9 to 8.5 months) [62]. Avelumab had anticancer effects in post hoc exploratory studies in high-risk populations, such as the elderly, patients with renal insufficiency, and patients with upper tract illness; patients with low albumin levels or liver metastases had significantly lower ORRs [62]. The objective response achieved within 3 months versus later was associated with longer OS (median not reached (95% CI 18.9 months to not evaluable) vs. 7.1 months (95% CI 5.2 to 9.0 months)). A total of 34.1% of patients showed PD-L1 positivity, 54.2% were PD-L1-negative, and 11.6% were not evaluable. In the PD-L1-positive cohort, the confirmed ORR was 23.8% (95% CI, 15.2–34.3%), whereas in the PD-L1-negative cohort it was 12.3% (95% CI, 7.2–19.2%). The median PFS in the PD-L1-positive cohort was 2.2 months (95% CI, 1.4–4.11%), while in the PD-L1-negative cohort it was 1.5 months (1.4–2.4%). However, the 12-month PFS rate in PD-L1-positive cases was 14.6% (95% CI, 8.8-21.9%) vs. 23.9% (95% CI, 14.2–35.0%) in PD-L1-negative ones. The median OS was 8.41 months (95% CI, 6.0–11.3%) in the PD-L1-positive cohort and 6.5 months (95% CI, 5.3–10.1%) in the PD-L1-negative one. The 24-month OS rate in PD-L1-positive cases was 24.3% (95% CI, 15.6–34.0%), compared to 17.9% (95% CI, 11.8–25.0%) in PD-L1-negative ones. Overall, PD-L1-positive patients showed a higher ORR, a longer median PFS and a better OS than PD-L1-negative ones [62]. Moreover, the safety analysis showed that 71.1% of patients had treatment-related adverse events of any degree, whereas 11.6% of them had grade 3–4 adverse events. One patient (0.4%) died as a result of treatment. The most prevalent immune-related adverse events (any grade) were immune-related rash (11.2%) and immune-related thyroid problems (13, 5.2%). Of note, 15.7% of patients stopped treatment because of its side effects. Consequently, the avelumab treatment demonstrated sustained effectiveness and tolerable safety in patients with platinum-treated advanced/metastatic urothelial carcinoma, including high-risk categories. Patients who responded within three months seemed to have a greater survival rate [62].

The second study, the phase 3 JAVELIN Bladder 100 trial, demonstrated that in patients with advanced urothelial carcinoma who were progression-free after first-line platinum-based chemotherapy, avelumab first-line maintenance plus best supportive care (BSC) significantly prolonged OS and PFS compared with BSC alone [63]. Patients were randomized 1:1 to receive either BSC alone (n = 350) or avelumab + BSC (n = 350). PD-L1 expression was assessed using the Ventana SP263 IHC PD-L1 assay (Ventana Medical Systems, Tucson, AZ, USA). PD-L1-positive status was defined if at least one of the following three criteria were satisfied: ≥25% PD-L1-positive tumor cells, ≥25% PD-L1-positive immune cells if >1% of the tumor area contained such cellular elements, or 100% PD-L1-positive immune cells if ≤1% of the tumor area contained immune cells. In the overall population, OS (primary endpoint) was significantly longer in the avelumab cohort than in the control one. OS at 1 year (measured from randomization) was 71.3% (95% CI, 66.0-76.0) in the avelumab cohort, while it was 58.4% (95% CI, 52.7 to 63.7) in the control one; the median OS was 21.4 months (95% CI, 18.9 to 26.1) and 14.3 months (95% CI, 12.9 to 17.9), respectively (stratified HR for death, 0.69; 95% CI, 0.56 to 0.86; repeated CI, 0.54 to 0.92; *p* = 0.001). Considering secondary endpoints, PFS was longer in the avelumab-treated patients compared to the control group in both primary populations. In the overall population, the median PFS was 3.7 months (95% CI, 3.5 to 5.5) in the avelumab cohort and 2.0 months (95% CI, 1.9 to 2.7) in the control one (stratified HR for disease progression or death, 0.62; 95% CI, 0.52 to 0.75). Overall, 51.1% had PD-L1-positive neoplasms in this study and the placebo and avelumab maintenance group showed balanced patients’ characteristics for the PD-L1-positive cohort. Avelumab significantly increased OS in PD-L1-positive patients: in fact, 1 year OS was 79.1% in the avelumab group and 60.4% in the control one (HR, 0.56; 95% CI, 0.40 to 0.79; *p* < 0.001) [63]. In the PD-L1-positive population, the median PFS was 5.7 months in the avelumab cohort and 2.1 months in the control one (HR, 0.56; 95% CI, 0.43 to 0.73). Adverse events from any cause showed an incidence of 98.0% in the avelumab cohort and 77.7% in the control one, while the incidence of adverse events ≥ grade 3 was 47.4% and 25.2%, respectively. 

Overall, the studies reported in our review show that ICIs are effective in the treatment of advanced bladder urothelial carcinoma and that PD-L1 status may or may not influence treatment response depending on several factors, such as the prior treatment history of patients and the immunotherapeutic agent used.

Table 2 provides an overview of different studies regarding PD-L1 evaluation in bladder carcinoma, including the different platforms used for immunohistochemical analysis, scoring systems, treatment choices, primary endpoints, and key findings from each study.

## 5. Conclusions

PD-L1 expression in advanced urothelial bladder carcinoma has important clinical implications for guiding therapeutic decisions and predicting treatment response. Targeting PD-L1 with ICIs can help restore the immune response against cancer cells, leading to enhanced antitumor immunity and potentially better outcomes. Indeed, high PD-L1 expression is often associated with a better response to PD-L1-targeted immunotherapy, and PD-L1 evaluation can improve treatment decisions, helping to identify the potential responders. However, it is important to note that not all patients with low or negative PD-L1 expression levels fail to respond to ICIs. As a matter of fact, ICIs may be prescribed in the second-line for advanced and metastatic urothelial carcinoma in cases of progression during or after platinum-based therapy, independent of PD-L1 status. Therefore, PD-L1 expression is not the exclusive determinant for the adoption of ICI therapy in bladder carcinoma. In fact, some other factors, such as tumor mutational burden, presence of tumor-infiltrating lymphocytes, molecular subtypes, and the overall tumor microenvironment, can also greatly influence the response to immunotherapy [64,65,66,67]. As a matter of fact, tumor cells can exhibit spatial and temporal heterogeneity in PD-L1 expression, meaning that a single biopsy may not capture the full spectrum of PD-L1 expression within a tumor. This heterogeneity can lead to discrepancies in predicting responses based on PD-L1 status [65]. Nonetheless, the tumor microenvironment can influence PD-L1 expression involving various factors, including the presence of immune cells, cytokines, and other signaling molecules. The dynamic nature of the tumor microenvironment can impact the predictive value of PD-L1 expression [65]. Additionally, alternative immune checkpoints and other regulatory pathways involved in modulating the immune response within the tumor microenvironment can influence the response to therapy independent of PD-L1 expression [65]. Moreover, tumor immune evasion mechanisms, such as upregulation of alternative immune checkpoints, recruitment of immunosuppressive cells, and secretion of immunosuppressive factors can counteract the effects of PD-L1 blockade and limit the efficacy of immunotherapy [67]. Furthermore, tumor genomic alterations, such as mutations affecting antigen presentation pathways or immune recognition, can impact the response to immunotherapy regardless of PD-L1 expression levels, thus influencing the tumor’s immunogenicity and susceptibility to immune-mediated killing [65]. Urothelial bladder carcinoma molecular subtypes can also influence PD-L1 expression and response to immunotherapy. In fact, basal molecular subtypes often exhibit increased immune infiltration and the upregulation of immune checkpoint molecules, including PD-L1 [67]. Similarly, squamous molecular subtypes are also associated with elevated PD-L1 expression [67]. In contrast, luminal molecular subtypes may show lower PD-L1 expression levels [67]. Altogether, molecular subtypes can influence the composition of the tumor microenvironment, including immune cell infiltration and cytokine profiles. Therefore, the molecular subtyping of bladder carcinoma can serve as a predictive biomarker for immunotherapy response and patients with specific molecular subtypes characterized by high PD-L1 expression and immune activation signatures may be more likely to benefit from immune checkpoint inhibitors [65,67]. Validating the utility of bladder molecular subtypes as predictive biomarkers for immunotherapy response requires large-scale clinical studies and integration with other biomarkers, such as tumor mutational burden (TMB), immune cell infiltration, and gene expression signatures. Of note, TMB plays a crucial role in PD-L1 expression and response to immunotherapy in advanced urothelial bladder carcinoma. In fact, high TMB levels in urothelial bladder carcinoma have been associated with increased PD-L1 expression. Tumors with elevated TMB tend to have a higher load of neoantigens, which are derived from mutated proteins and can trigger an immune response. This increased immunogenicity, coupled with PD-L1 expression, creates a favorable tumor microenvironment for ICI therapy [64]. TMB is not a static parameter and can evolve over the course of treatment or disease progression. Monitoring changes in TMB levels and adapting treatment strategies accordingly may be necessary to maximize the benefits of immunotherapy [64,67]. Another key factor influencing PD-L1 expression and response to immunotherapy in advanced urothelial bladder carcinoma is represented by epigenetic modifications, such as DNA methylation, histone modifications, and non-coding RNAs [68]. In fact, aberrant DNA methylation patterns have been associated with bladder cancer development and progression [68]. Hypermethylation of the *PD-L1* gene promoter region can lead to silencing of PD-L1 expression, while hypomethylation may result in increased PD-L1 expression. Interestingly, specific genes such as *hTERT*, *TWIST1*, *VIM*, and *NID2* have been shown to be promising tools for DNA methylation in bladder cancer [68]. Moreover, circulating tumor DNA and RNA in body fluids, such as urine, serum, and plasma, are being investigated as noninvasive biomarkers for early detection and monitoring of bladder carcinoma [68], and mainly DNA methylation analysis should be proposed as diagnostic support for the diagnosis of urothelial carcinoma in urinary samples [3,4,5,6,7,8]. Furthermore, histone modifications, including acetylation and methylation, have shown an impact on *PD-L1* gene expression by altering chromatin structure and gene accessibility, and changes in histone marks at the *PD-L1* locus have shown an influence on its transcriptional activity. Lastly, non-coding RNAs, such as microRNAs and long non-coding RNAs, can post-transcriptionally regulate PD-L1 expression by binding to mRNA and modulating its stability or translation, and their dysregulation can affect PD-L1 levels in cancer cells. All of these findings could have an important clinical impact; in fact, targeting epigenetic regulators involved in PD-L1 expression could potentially enhance the efficacy of immunotherapy in urothelial bladder carcinoma. The drugs that modulate DNA methylation or histone modifications may be used in combination with ICIs to overcome resistance mechanisms. Moreover, strategies to account for intra-tumor heterogeneity and monitor dynamic changes in epigenetic profiles over time would be needed. In fact, identifying patient-specific epigenetic signatures associated with ICI treatment response could allow the optimization of immunotherapy strategies in advanced urothelial bladder carcinoma. Overall, epigenetic biomarkers could be used to stratify patients based on their likelihood of responding to ICI therapy, and their integration with traditional biomarkers like PD-L1 could improve patient selection and treatment outcomes. The integration of such biomarkers into personalized therapeutic approaches could provide a more holistic understanding of each patient’s tumor biology and immune response, leading to more personalized and effective treatment strategies.

## Figures and Tables

**Table 1:** Different PD-L1 scoring systems commonly used in bladder carcinoma.

Scoring System	Description	Platform Used	Positivity Criteria
**CPS**	Ratio of PD-L1 stained cells (tumor cells, lymphocytes, macrophages) to total viable tumor cells, multiplied by 100	Dako Agilent 22C3 platform	CPS > 10
**TPS**	Percentage of tumor cells showing PD-L1 expression	Various platforms	TPS cut-off varies
**IC Score**	Area occupied by PD-L1-positive immune cells (lymphocytes, dendritic cells, macrophages, granulocytes) as a percentage of whole tumor area	Ventana PD-L1 SP142 platform	IC score of 2/3

Abbreviations: CPS, combined positive score; TPS, tumor proportion score; IC score, immune cell score.

**Table 2:** Different studies regarding PD-L1 evaluation in bladder carcinoma, including the platforms used for immunohistochemical analysis, scoring systems, treatment choices based on PD-L1 expression, and key findings from each study.

PD-L1 Expression	Study Approval	PD-L1 Cut-Off and Assay	Target Drug	Patients	Primary Endpoint(s)	Study Findings
PD-L1 status not important	KEYNOTE-045	CPS ≥ 10 (Dako 22C3 assay)	Pembrolizumab	Second-line for advanced and metastatic urothelial carcinoma that progresses during or after platinum-based therapy	The coprimary endpoints were OS and PFS, which were assessed among all patients and among patients who had a tumor PD-L1 CPS ≥ 10	Pembrolizumab was associated with significantly longer OS and a lower risk of treatment-related adverse events than chemotherapy
PD-L1 status not important	IMvigor211	IC score of 0, 1, 2/3 (Ventana SP142 assay)	Atezolizumab	Second-line for advanced and metastatic urothelial carcinoma that progresses during or after platinum-based therapy	OS was the primary endpoint	Although the primary analysis did not demonstrate statistically significant longer OS for patients receiving atezolizumab versus chemotherapy, the updated OS revealed long-term durable remission. With a median follow-up of 33 months, ITT patients receiving atezolizumab had better OS rates at 24 months and 30 months, as well as across PD-L1 subgroups, than those receiving chemotherapy
PD-L1 status not important	KEYNOTE-052	CPS ≥ 1 and CPS ≥ 10 (Dako 22C3 assay)	Pembrolizumab	First-line for cisplatin-ineligible patients with locally advanced and unresectable or metastatic urothelial carcinoma	ORR was the primary endpoint	A PD-L1 CPS ≥ 10 was associated with a higher frequency of response to pembrolizumab. However, responses were observed in all PD-L1 expression categories
PD-L1 status not important	IMvigor210	IC score of 0, 1, 2/3 (Ventana SP142 assay)	Atezolizumab	Patients with locally advanced or metastatic urothelial carcinoma who were not eligible for cisplatin	The main outcome was the independently verified ORR	All PD-L1 and poor prognostic factor subgroups showed responses
PD-L1 status not importantt	ABACUS	IC score of 2/3 (Ventana SP142 assay)	Atezolizumab	Neoadjuvant setting of patients with MIBC, who were ineligible for or refused cisplatin-based neoadjuvant chemotherapy	The primary clinical endpoint was the pCR rate in all patients who received at least one cycle of atezolizumab and underwent radical cystectomy, or withdrew from the study for disease progression before surgery	Neoadjuvant atezolizumab was associated with both improved clinical responses and DFS
PD-L1-positive tumor	KEYNOTE-361	CPS > 10 (Dako Agilent 22C3 assay)	Pembrolizumab	First-line therapy for metastatic/advanced urothelial carcinoma cisplatin-unfit patients	The dual primary endpoints were PFS and OS for pembrolizumab plus chemotherapy versus chemotherapy alone for the total patient population	There was no significant difference in PFS between the groups receiving pembrolizumab plus chemotherapy and chemotherapy alone
PD-L1-positive tumor	IMvigor130 trial	IC score of 2/3 (Ventana SP142 assay)	Atezolizumab	First-line therapy for metastatic/advanced urothelial carcinoma cisplatin-unfit patients	The co-primary endpoints were investigator-assessed PFS and OS for group A against group C in the ITT population (i.e., all randomly assigned patients), as well as OS for group B versus group C, evaluated hierarchically, and then the populations with PD-L1 IC2/3 if the results from group A versus group C were significant	Overall, the first-line combination of atezolizumab and platinum-based chemotherapy did not significantly increase OS in the ITT group, while improving PFS rates. An OS improvement was observed among PD-L1 IC2/3 patients treated with atezolizumab monotherapy
PD-L1-positive tumor	CheckMate 274 trial	TPS ≥ 1% (Dako Agilent 28-8 pharmDx assay)	Nivolumab	Adjuvant treatment after radical cystectomy for urothelial carcinoma patients at high risk of recurrence, and PD-L1-high-expressing high-risk patients	The two primary end points were DFS among all the patients who underwent randomization (ITT population) and among those with a TPS ≥ 1%	Globally, adjuvant nivolumab was associated with a longer DFS than placebo both in the ITT population and among patients with a PD-L1 TPS ≥ 1%. Conversely, no appreciable effect was observed in patients with negative PD-L1 expression (TPS < 1%) when nivolumab was administered compared to placebo
PD-L1-positive tumor	JAVELIN Solid Tumor trial	TPS ≥ 5% (Dako Agilent 73-10 pharmDx assay)	Avelumab	Patients with platinum-treated advanced/metastatic urothelial carcinoma	Best overall response, PFS, OS, and safety were the endpoints	Avelumab demonstrated sustained effectiveness and tolerable safety in all categories. Patients who responded within three months seemed to have a greater survival rate. Altogether, PD-L1-positive patients showed a higher ORR, a longer median PFS, and a better OS than PD-L1-negative ones
	JAVELIN Bladder 100 trial	PD-L1-positive status was defined if at least one of the following three criteria were satisfied: ≥25% PD-L1-positive tumor cells, ≥25% PD-L1-positive immune cells if >1% of the tumor area contained such cellular elements, or 100% PD-L1-positive immune cells if ≤1% of the tumor area contained immune cells (Ventana SP263 assay)	Avelumab first-line maintenance plus BSC	Patients with advanced urothelial carcinoma who were progression-free after first-line platinum-based chemotherapy	The primary end point was OS	Avelumab first-line maintenance plus BSC significantly prolonged OS and PFS compared with BSC alone. Avelumab significantly increased OS in PD-L1-positive patients and in the PD-L1-positive population, the median PFS was higher in the avelumab cohort than in the control one

Abbreviations: CPS, combined positive score; TPS, tumor proportion score; IC score, immune cell score; ITT, intention-to-treat; ORR, objective response rate; MIBC, muscle-invasive bladder cancer; pCR, pathological complete response; DFS, disease-free survival; BSC, best supportive care; PFS, progression-free survival; OS, overall survival.

## Data Availability

No new data were created or analyzed in this study.
